# Prevalence of premature ovarian insufficiency (POI) and its relationship with female reproductive factors in Iranian women: a cross-sectional study from the Persian (Shahedieh) cohort data

**DOI:** 10.1186/s12905-023-02620-9

**Published:** 2023-09-01

**Authors:** Sara Jambarsang, Mahsa Khodayarian, Reyhane Sefidkar, Nooshin Yoshany

**Affiliations:** 1 Center for Healthcare Data Modeling, Departments of Biostatistics and Epidemiology, Shahid Sadoughi University of Medical Sciences, Yazd, Iran; 2 Department of Health Education and Health Promotion, Social Determinants of Health Research Center, School of Public Health, Shahid Sadoughi University of Medical Sciences, Yazd, Iran

**Keywords:** Lactation, Early menopause, Parity, Premature ovarian failure

## Abstract

**Background:**

In premature ovarian insufficiency, the cessation of menstruation, and cessation of ovarian function occurs before the age of 40, and this phenomenon is associated with many complications and problems for women. Since several factors can affect this situation, this study was conducted to determine the relationship between fertility history, and premature ovarian failure.

**Methods:**

This cross-sectional study was conducted on the data of the first phase of cohort study, which was a sample of 10,000 people from an Iranian adult population (age: 35–70 years). 1276 women were included who naturally experienced menopause from this population. They were separated into three groups based on the age of menopause: premature ovarian failure for those who reached menopause before the age of 40, early menopause for those who reached menopause between the ages of 40 and 45, and natural menopause for those who reached menopause at or after the age of 45. The demographic and fertility characteristics of two groups of women, one with premature ovarian failure and the other with early menopause, were compared with a group of women experiencing normal menopause. The comparison was based on frequency and percentage. Moreover, the odds ratio (OR) of these two groups compared to normal group was crudely calculated, and adjusted based on age at the time of the interview using a logistic regression model. SPSS 23 software was used to fit models and calculations.

**Results:**

The prevalence of premature ovarian failure was 3%. The likelihood of premature ovarian failure decreases as the number of live births rises. The risk is considerably higher for births ranging from zero to three children compared to those with more than four. Increased duration of breastfeeding is associated to a reduced risk of premature ovarian failure compared to the spontaneous occurrence (OR = 0.98, 95% CI (0.97, 0.99)). This relationship is maintained even after adjusting for age (OR = 0.98, 95% CI (0.97, 0.99).

**Conclusion:**

Based on the results of present study, it can be concluded that the factor of the number of births, and the duration of breastfeeding affect reducing the occurrence of POI, therefore, in health and treatment programs and policies, encouragement to have children, which is now part of the policies population of Iran, and the importance, and benefits of breastfeeding for mother and baby should be emphasized more.

## Introduction

Menopause time depends on ovarian function, and normal menopause is equal to 12 consecutive months of amenorrhea, without factors, such as hysterectomy, oophorectomy, chemotherapy, radiotherapy, etc. [1]. Every woman’s reproductive aging process is marked by a gradual decline in both the quantity and quality of ova within the ovarian follicles. The age at which menopause occurs is influenced by several factors, including the initial number of ova a woman is born with, the rate at which ova are lost over her lifetime due to the atresia process, and the minimum threshold of ova required to sustain hormone production for menstruation to continue [2–5]. 21 studies in 10 countries (high-income countries) showed that the average age of natural menopause is between 47 and 53 years. This is even though the age of menopause can be different in low, and middle-income countries [6]. Menopause before the age of 40 is commonly known as premature menopause, although premature ovarian failure (POF) is currently considered the most appropriate term to indicate the loss of ovarian function because it does not specify definitive failure. Gonadotropin levels, especially FSH, must be more than or equivalent to 25 IU/L on two consecutive occasions in less than four weeks in order to be used to diagnosis premature menopause [7, 8]. In usual, the estimations obtained from the results of the studies indicate that the prevalence of premature ovarian failure ranges from 0.9 to 2% [7, 9–11]. The prevalence of POI in Iranian population (menopause age < 40 years), and early menopause (menopause age < 45 years) was reported as 3.5% and 24.6%, respectively [12]. Menopause between the ages of 40 and 45 is referred to as early menopause. Premature menopause is present in 2% of women in high-income nations while early menopause is seen in 7.6% [11]. Women’s life expectancy and lifespan have grown due to the decline in infectious illness mortality, and as a result, more years of their lives will be spent going through menopause. Hot flashes, osteoporosis, diminished sexual drive, and other issues are common among menopausal women [13]. Premature menopause increases the incidence of cardiovascular diseases, and osteoporosis, in other words, for each year of delay in menopause, 2% of the mortality rate due to cardiovascular diseases decreases [14]. The causes of POI are broken down into six categories in the recommendations of European Society of Human Reproduction and Embryology (ESHRE), including chromosomal or genetic problems, autoimmune illnesses, infections, iatrogenic causes, environmental, and idiopathic factors [15]. Premature menopause is often idiopathic, but there are some genetic and autoimmune causes, the most common of which are defects related to X chromosome. Only a few epidemiological studies have particularly looked at the connection between early or premature menopause and non-genetic variables [16–18]. Smoking and alcohol use are two environmental variables that contribute to POI [15]. Smoking is associated with an increase in the risk of premature menopause, while regular physical activity (one to several times a week), and drinking moderate amounts of alcohol (one to three times a month) are associated to a decrease in the risk of premature menopause [19]. This study’s results conducted in America indicated that breastfeeding is associated to higher levels of AMH (anti-Müllerian hormone), and later onset of menopause [20]. Extended breastfeeding is linked to a lower risk and the incidence of vasomotor episodes after menopause [21]. A research found that the age of menopause was influenced by various variables, including education, the number of pregnancies, nursing, and using birth control pills [22]. In a study conducted in Massachusetts, no relationship was found between the studied factors and the age of menopause [23]. In another study, only factor related to the age of menopause was spouse’s occupation [24]. This research was carried out to identify the variables impacting early menopause in light of the existence of contradictions and many factors affecting the start of menopause.

## Methods

### Study design and participants

This cross-sectional research used information from PERSIAN cohort study’s recruiting phase, which included a representative sample of adult Iranians (aged 35 to 70). Its name is the Shahedieh cohort study. For the Shahedieh cohort study, about 10,000 adults living in three cities of Yazd Greater Area (Shahedieh, Zarch, and Ashkezar), located in Yazd Province, Iran, and from the year 2016 to present were recruited. Detailed information about the protocol of PERSIAN cohort study is provided elsewhere [25].

**Fig. 1:**
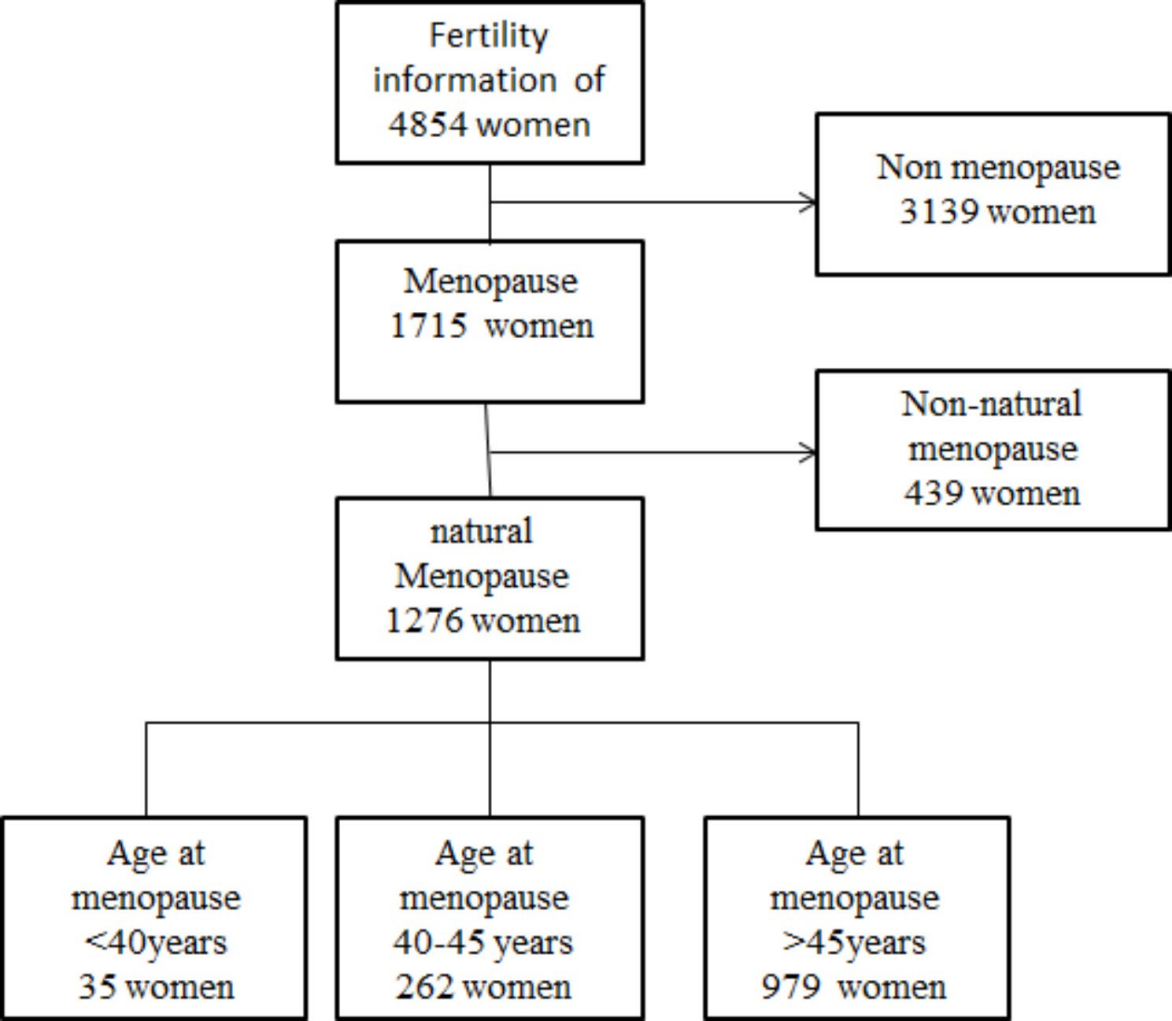
The flowchart of women in the study

Fertility information of 4854 women is recorded in this sample. Among them, 1715 people had gone via the menopause, and 1276 people had gone through menopause spontaneously. All women who experienced menopause naturally were included. Based on the age of menopause, they were classified into three categories: premature ovarian failure for people who experienced menopause before 40 years old, early menopause for women with menopausal age between 40 and 45 years old, and normal menopause with the menopausal age over 45. The details of this sample are shown in Fig. 1.

Demographic and fertility data of the women included in the study were extracted from the cohort database, including their age, level of education, employment status, menarche age, use of infertility medications, number of live births, use of oral contraceptives, age of first delivery, number of pregnancies, and number of months of breastfeeding.

### Statistical analysis

Demographic and fertility characteristics of women in two groups with the premature ovarian failure, and premature menopause were compared due to frequency and percentage with the group of women with normal menopause experience. Moreover, the odds ratio (OR) of these two groups compared to normal group was crudely calculated, and adjusted based on age at the time of the interview. Adjustment for age at the interview was made because younger people tend to have fewer children in terms of the cultural changes. Using unconditional multiple logistic regression (MLR), fitted by the technique of maximum likelihood, Table 1 shows the raw and adjusted OR of premature ovarian failure and early menopause as well as the related 95% confidence intervals (CI). To study the duration of breastfeeding with menopause status, three logistic models were considered in different numbers of live births: First, the model includes only the number of months of breastfeeding. The second model included the months of breastfeeding and age at the interview. The third model included the mother’s age at the time of the first birth in addition to the factors from the first model. Variables included in the models are shown in a footnote to Table 2. SPSS 23 software was used to fit models and calculations.

**Table 1:** Distribution of cases with spontaneous menopause at age < 40 and 40–45 years, and controls with age at menopause > 45 years according to selected factors

	No. of cases		controls	OR(95% CI)		adjusted OR (95% CI)**	
	Age at menopause < 40years	Age at menopause 40–45 years	Age at menopause >45years	Age at menopause < 40years	Age at menopause 40–45 years	Age at menopause < 40years	Age at menopause 40–45 years
**Education**							
illiterate	18(51.4)	153(58.4)	574(58.6)	1.33(0.65,2.69)	1.34(0.68,2.63)	1.52(0.71,3.26)	1.03(0.75,1.42)
literate	17(48.6)	109(41.6)	405(41.4)	1+	1+	1+	1+
**Age at menarche (years)**							
≤ 11	2(5.7)	23(8.8)	79(8.1)	0.55(0.13,2.37)	1.15(0.69,1.90)	0.43(0.09,1.87)	1.14(0.69,1.89)
12	2(5.7)	44(216.8)	139(14.2)	0.31(0.07,1.34)	1.25(0.85,1.85)	0.26(0.06,1.11)	1.24(0.84,1.84)
13	7(20.0)	63(24.0)	237(24.3)	0.64(0.27,1.51)	1.05(0.75,1.47)	0.55(0.23,1.32)	1.05(0.74,1.47)
≥ 14	24(68.6)	132(50.4)	522(53.4)	1+	1+	1+	1+
**Use Infertility Drug**							
Yes	4(11.4)	15(5.7)	51(5.2)	2.35(0.79,6.90)	1.10(0.61,1.99)	1.93(0.64,5.81)	1.09(0.60,1.98)
No	31(88.6)	247(94.3)	928(94.8)	1+	1+	1+	1+
**Alive Childbirth No.**							
0	4(11.4)	6(2.3)	13(1.3)	15.24(4.50,51.57)	1.82(0.68,4.83)	12.21(3.49,42.64)	0.99(0.97,1.02)
1	2(5.7)	5(1.9)	13(1.3)	7.62(1.59,36.41)	1.51(0.53,4.29)	5.42(1.08,27.23)	1.81(0.68,4.83)
2	3(8.6)	9(3.4)	28(2.9)	5.31(1.47,19.16)	1.26(0.59,2.72)	3.87(1.03,14.46)	1.51(0.53,4.30)
3	9(25.7)	28(10.7)	82(8.4)	5.44(3.35,12.58)	1.34(0.85,2.12)	3.90(1.59,9.56)	1.34(0.84,2.15)
≥ 4	17(48.6)	214(81.7)	842(86.1)	1+	1+	1+	1+
Mean(sd)	4.29(3.06)	5.86(2.78)	6.01(2.53)	Trend p-value < 0.001	Trend p-value = 0.07	Trend p-value < 0.001	Trend p-value = 0.08
**Oral contraceptive use**							
No	23(65.7)	156(59.5)	522(53.3)	1.68(0.83,3.41)	1.29(0.98,1.69)	1.94(0.95,3.98)	1.30(0.99,1.72)
Yes	12(34.3)	106(40.5)	457(46.7)	1+	1+		
**Has Job**							
No	30(85.7)	246(93.9)	928(94.8)	0.33(0.12,0.88)	0.84(0.47,1.51)	0.50((0.18,1.39)	0.86(0.48,1.55)
Yes	5(14.3)	16(6.1)	51(5.2)	1+	1+	1+	1+
**Age at First Alive Childbirth**							
> 18 year	7(22.6)	74(28.9)	322(33.4)	0.58(0.25,1.36)	0.81(0.59,1.09)	0.59(0.25,1.39)	0.81(0.59,1.09)
18–35 year	24(77.4)	180(70.3)	634(65.8)	1+	1+	1+	1+
> 35 year	0	2(0.8)	7(0.7)	*		*	
**Breastfeeding duration**							
Mean(sd) Trend p-value = 0.002	69.16(39.7)	96.71(49.6)	101.55(52.3)	0.98(0.97,0.99)	0.99(0.98,1.01)	0.98(0.98,0.99)	1.01(0.99,1.02)

Odds ratios were estimated using the unconditional multiple logistic regression (MLR).

*Merge into the second category to fit the regression model because of low sample size.

** Adjusted for age at interview.

**Table 2:** Multivariable Associations of breastfeeding duration with Menopause status in Iranian women stratified on the number of live birth

	OR(95% CI) model 1		OR(95% CI) model 2		OR(95% CI) model 3	
the number of live birth (No.)	Age at menopause < 40years	Age at menopause 40–45 years	Age at menopause < 40years	Age at menopause 40–45 years	Age at menopause < 40years	Age at menopause 40–45 years
1 (20)	0.87(0.74,1.02)	0.90(0.80,1.01)	0.86(0.70,1.05)	0.91(0.81,1.02)	0.84(0.65,1.08)	0.91(0.80,1.02)
2 (40)	1.03(0.96,1.05)	1.00(0.96,1.05)	1.03(0.96,1.05)	1.00(0.96,1.05)	0.96(0.87,1.05)	0.99(0.93,1.06)
3 (119)	1.01(0.98,1.05)	1.00(0.99,1.03)	1.01(0.98,1.05)	1.00(0.99,1.03)	1.01(0.97,1.05)	1.01(0.99,1.03)
4 (188)	0.99(0.96,1.02)	1.00(0.99,1.01)	0.99(0.96,1.02)	1.00(0.99,1.01)	0.98(0.95,1.02)	1.00(0.99,1.02)
≥ 5 (885)	0.99(0.98,1.00)	0.99(0.99,1.00)	0.98(0.97,0.99)	0.99(0.99,1.00)	0.98(0.97,0.99)	0.99(0.99,1.00)

Odds ratios were estimated using unconditional multiple logistic regression (MLR).

Model 1: crud model of the association between breastfeeding duration, and menopause status; reference group is the women with age at menopause > 45 years.

Model 2: Adjusted for age at the interview.

Model 3: Adjusted for age at interview, age at menarche, and first alive birth child age.

## Results

Among 1276 women with the spontaneous menopause who entered the recruitment phase of this cohort, 35 (3%) reported premature ovarian failure, and 262 (20%) spontaneous menopause at age 40–45 years, 979(77%) women declared that they went via the menopause after the age of 45. Table 1 shows their distribution and those with menopause at age > 45 years based on selected factors.

Premature ovarian failure is less likely to occur if there are more children born. Having less than four children dramatically reduces this risk, whereas having more than four children greatly increases it. For example, the risk of premature ovarian failure in the people with 3 children is five times (OR = 5.44, 95% CI (3.35, 12.58)) that of people with 4 or more children. This risk persisted even after adjusting for age at the interview (OR = 3.90, 95% CI (1.59, 9.56)) (Table 1).

Increasing the duration of breastfeeding is associated to reducing the risk of premature ovarian failure compared to its spontaneous occurrence (OR = 0.98, 95% CI (0.97, 0.99)). This relationship holds even after adjusting for age at the interview (OR = 0.98, 95% CI (0.98, 0.99)). However, no significant difference was observed for the risk of premature menopause over the age of 45 based on the amount of breastfeeding.

No significant association emerged among the odds of premature ovarian failure or menopause at age 40–45 and education, Job, age at menarche, oral contraceptive use, First Alive Childbirth Age, or Use of Infertility Drugs (Table 1).

In order to undertake a more detailed study of the relationship between breastfeeding length and menopausal state, the data were separated into groups based on the number of live births from one to four and more than and equal to five children. In this classified analysis, based on three different models, the risk of premature ovarian failure, and premature menopause was calculated compared to its normal state. The results showed that the risk of premature ovarian failure is lower only after adjusting for age (OR = 0.98, 95% CI (0.97, 0.99)), age at menarche, and age at first birth (OR = 0.98, 95% CI (0.97, 0.99)) in the people with five or more children.

## Discussion

This study found that the number of parity, and breastfeeding was associated to a decreased risk of premature ovarian failure. No relation emerged with Job, age at menarche, oral contraceptive use, First Alive Childbirth Age, or Use Infertility Drugs. Similar findings emerged when we compared women with menopause at age 40 to < 45 and women with the menopause at ≥ 45 years.

3% of women had premature menopause, 20% had early menopause among the ages of 40–45, and 77% had menopause over 45 years of age. In a study conducted in Iran, the prevalence of POI was 3.5% and early menopause was 24.6% [26], which is close to the prevalence found. The geographic, cultural, and genetic closeness of Iranian people may be the cause of this resemblance. According to a study conducted there in 2017 [26], 5.5% of women had early menopause, with one Indian hamlet having the highest prevalence at 14.6%. Rural women are more likely to experience premature menopause in terms of poor access and health care [27]. This study’s results conducted in 2021 in India showed that the percentage of premature menopause is 3.7%, of which 1.2% of them were spontaneous (natural menopause), and 1.7% of them experienced premature menopause in terms of surgeries, such as hysterectomy and oophorectomy [28]. In the study conducted in Korea and Shanghai, the prevalence of premature menopause was 2.41% and 2.8%, respectively [15, 26], which is lower than in Iran, and this inconsistency can be related to the differences in people’s lifestyles.

Even if the current research revealed no link between early menopause and education, this is still true. They are more likely to seek medical services since educated individuals often take better care of themselves and pay more attention to their health. In a study, it was shown that education has a significant and inverse relationship with early menopause, which means that by increasing the level of education, the rate of early menopause decreases [26]. In two studies conducted in Korea, the prevalence of POI was significantly higher in the people with lower household incomes, and lower education levels [15, 29]. The observed inconsistency can be in terms of cultural differences, and the level of access to health services. It seems that there is a noticeable difference between educated and illiterate individuals in terms of follow-up and access to health facilities in a nation like India, where the health and treatment situation is quite poor [30], but in Iran and Yazd province, which is one of the poles. It is considered medicine and therapy and most people have proper access to health services, there is no significant difference between educated and illiterate people.

There was no relationship between being employed and not employed and premature menopause. In other studies, it was reported that housewives had more chance of early menopause [26, 27]. This discrepancy may be attributable to the nature of study, the regional cultural context, and the low employment rate among women in the region where the study was conducted. All women were married and other factors, such as the number of births and duration of breastfeeding had a significant relationship with the occurrence of early menopause. The age at which menopause begins did not significantly correlate with marital status in a research done in Greece [31]. According to a different research, divorced and bereaved women are more likely to have early menopause [27, 32]. Ovulation ceases during pregnancy, therefore the more births and pregnancies a woman has, the more eggs she retains, which helps prevent early menopause in women. Our study’s findings (women having a history of the deliveries of at least five babies considerably Compared to other women, it was less likely to have early ovarian failure. The results of a cool study showed that the women had a high risk for premature menopause (both pre-wrestle and Early) [26]. Another study from India and USA was reported that the higher the pregnancy, the less risk for early menopause [27, 33]. In the study conducted in Italy, parity was related to a reduced risk of POF, and this reduction increased with the number of life births; compared to the nulliparous, the OR of POF was 0.4, 0.5, and 0.2 in women with 1, 2, and 3 or more children, respectively [34].

This study’s results are not consistent with the results of previous studies that showed that the age of menarche, and the age of the first pregnancy are strong predictors of the onset of menopause [26, 27, 35, 36]. In aforementioned studies, it was reported that the younger the menarche age, the higher the risk of premature menopause. Various study settings, a lack of confounder correction, a lack of statistical power, and the fact that many of these studies use clinical-based samples of women rather than samples derived from the general community might all contribute to this disparity. It is caused by the context of studied society that the first birth of a child over the age of 35 occurred in a very small number of people, so this relationship was not observed. The culture of our study area encourages people to get married, and get pregnant among the ages of 18 and 35. It seems that women who had premature menarche have less ovarian reserve and therefore premature menopause is more likely to occur.

This study’s results showed that women who had a longer duration of breastfeeding had a lower chance of premature menopause, which is consistent to the findings of another study in Korea [37]. Its justification can be that women usually do not ovulate during breastfeeding, the longer the duration of breastfeeding, the more eggs they store and the less likely they are to experience premature menopause.

The limitations of the research should not be overlooked. Since this study relies on previously published material, the authors did not verify the data gathering process or its correctness. On the other hand, because this study was conducted based on the data of a small part (Shahedieh, Zarch, and Ashkezar) of Yazd province, its information cannot be generalized to the whole of Yazd province and the country of Iran. Furthermore, the factors related to people’s previous lifestyle (before menopause), such as physical activity, nutrition, alcohol consumption, and smoking could not be measured in terms of the type of study, so only the factors related to the obstetric history of people whose information was available were examined.

## Conclusion

Based on the results of the present study, it was seen that women with higher parity and more history of breastfeeding are less exposed to the early menopause; the present results can be used as a basis for interventional and forward-looking research. It is suggested to conduct longitudinal studies considering other fertility variables, as well as lifestyle variables. In order to reduce their risk of early menopause and avoid Iran from witnessing an aging demographic disaster in the not-too-distant future, women should be urged to have children and breastfeed. Shahedieh cohort study was conducted based on the guidelines laid down in Declaration of Helsinki and informed consent was obtained from participants or legally authorized representatives of illiterate participants.

## Data Availability

The datasets used and/or analyzed during the current study are available from the corresponding author on reasonable request.
